# Age-related limitations of interleukin-6 in predicting early mortality in acute ST-elevation myocardial infarction

**DOI:** 10.1186/s12979-014-0023-7

**Published:** 2014-12-04

**Authors:** Dominika Kanikowska, Małgorzata Pyda, Katarzyna Korybalska, Stefan Grajek, Maciej Lesiak, Andrzej Bręborowicz, Janusz Witowski

**Affiliations:** Department of Pathophysiology, Poznan University of Medical Sciences, Rokietnicka 8, 60-806 Poznań, Poland; 1st Department of Cardiology, Poznan University of Medical Sciences, Poznan, Poland

**Keywords:** Aging, Interleukin-6, Mortality, Myocardial infarction

## Abstract

Interleukin-6 (IL-6) is an inflammatory cytokine whose levels increase significantly during myocardial infarction (MI).

It has been hypothesised that the concentrations of IL-6 at admission may be useful in prognosticating long-term outcomes. It is unclear, however, whether IL-6 could improve the prognosis of early mortality in MI.

We have compared serum IL-6 levels and analysed the disease course in 158 patients with ST-elevation MI (STEMI) who either survived (n = 148) or died (n = 10) within 30 days following the admission. Patients were treated in a single university centre with primary percutaneous coronary intervention (PCI).

The non-survivors (6.3%) displayed most of typical risk factors for poor outcome. In addition they had significantly higher concentrations of IL-6 at hospital admission (median values 8.5 vs. 2.0 pg/ml; p = 0.038). However, they were also significantly older than the survivors (median values 72 vs. 57 years; p = 0.0001). IL-6 levels are known to increase with age and we could confirm a significant correlation between patients’ calendar age and circulating IL-6 (p = 0.009). Regression analysis revealed that IL-6 concentrations were significantly affected by patients’ age but they did not independently relate to patients’ outcome.

Such results indicate that circulating IL-6 at admission may be of limited value in predicting early mortality in STEMI. It is important to recognize that, because of the small group of patients who died (N = 10), the results must be interpreted with caution. Therefore, we stress that these results should be viewed as preliminary and further validated in a larger set of patients.

## Introduction

IL-6 is a key mediator of inflammation [1,2] and as such has been implicated in the pathogenesis of atherosclerosis and coronary artery disease (CAD) [3]. Biological effects of IL-6 are mediated either by classic IL-6 receptor (IL-6R) or by trans-signalling via soluble IL-6R (sIL-6R) [4]. Positive associations between circulating IL-6 concentrations and the risk of CAD have been consistently observed [5]. Mendelian randomization studies indicate there might exist causal association between signalling through IL-6R and CAD [6].

Moreover, results of several studies suggest that high IL-6 levels during acute coronary syndromes (ACS) may be associated with worse long-term prognosis [7-13]. Less clear is the link between IL-6 and early mortality in ACS. On the one hand, high IL-6 was proposed to predict 30-day mortality in patients with cardiogenic shock after myocardial infarction [14,15]. On the other hand, the association of high baseline IL-6 concentrations with increased mortality within 16 weeks after ACS became insignificant when adjusted for known covariates [9].

Chronologic age is a strong independent risk factor for increased morbidity and mortality from ACS [16,17]. The role of age as a covariate may be of particular importance in interpreting IL-6 data. It is because ageing is associated with a rise in circulating IL-6, which may be related both to an increased prevalence of cardiovascular risk factors and to ageing *per se* [18,19]. The relative contribution of these process is difficult to estimate and differs probably between the populations studied.

We thought we could add to this debate by examining if serum levels of IL-6 recorded at admission predict 30-day mortality in patients with myocardial infarction. Experimentally, such a study would require groups of patients who had been controlled for age and other risk factors, including duration of CAD. However, in practice, this would be very difficult to achieve. Accordingly, the effect of such factors has been taken into account statistically.

## Material and methods

Samples from 158 patients with acute ST-elevation myocardial infarction (STEMI) were analysed. Blood samples were taken in the acute phase of STEMI on admission to the hospital and before primary PCI. The diagnosis was based on typical clinical, biochemical, and electrocardiographic criteria. All patients underwent primary percutaneous coronary intervention (PCI) within 12 hours after the onset of chest pain. The procedure was performed according to standard institutional practices with stenting whenever possible. After PCI the patients were maintained on standard dual anti-platelet therapy (aspirin, clopidogrel), β-blockers, and angiotensin-converting enzyme inhibitors. Statins (atorvastatin or simvastatin) were introduced 48 hours after PCI. Patients with pre-existing autoimmune or inflammatory disease, malignancy, immunosuppression, and prior therapy with statins were not included in the analysis, as these conditions may independently affect IL-6 levels.

IL-6 and sIL-6R were measured with enzyme-linked immunosorbent assay using DuoSet Development kits (R&D Systems, USA). The assays were performed as per manufacturer’s instructions. Sensitivity of the assays, as calculated by adding two standard deviations to the mean read-out for blank samples (n = 16), was 2 pg/ml and 9 pg/ml for IL-6 and sIL-6R, respectively. To enable logarithmic transformation, values below the limit of detection were assigned a value of 0.1 rather than zero.

Statistical analyses were performed using SPSS v.20 software (IBM, USA). The comparisons were made between patients who either survived or died within 30 days after STEMI. The normally distributed data were analysed using t-tests and Pearson’s correlation; the data that were not distributed normally were compared using Mann-Whitney’s U-test and Spearman’s correlation. For regression analysis, the abnormally distributed data were log transformed. In this analysis, possible confounders (age and length of time before the cardiac event that CAD had been experienced) were incorporated in order to focus upon the issue of whether or not the variables of interest were associated with patient survival. Categorized data were analysed with the Fisher’s test. The level of significance was set at P < 0.05.

### Ethical approval

Ethics Committee of the Poznan University of Medical Sciences.

## Results

Of 158 STEMI patients analysed, 10 (6.3%) died within 30 days. Eight deaths occurred during initial hospitalization and were related to either cardiogenic shock (7 patients) or ventricular fibrillation (1 patient). The characteristics of patients from the two groups are listed in Table 1. Mean time from the onset of pain to PCI (total ischemic time) in survivors was 4.2 +/− 2.6 hours and in non-survivors was 7.1 +/− 2.0 hours (p < 0.001).

**Table 1:** **Patient characteristics**

	**Survivors (n = 148)**	**Non-survivors (n = 10)**	**P**
*Demographic parameters*			
Age, yrs	57 (50–64)	72 (68–80)	0.0001
Gender (men,%)	70	72	1.00
BMI (kg/m2)	26.0 (24.1-28.0)	25.0 (21.0-26.8)	0.27
*Medical history*			
Hypertension,%	45	45	1.00
Diabetes,%	13	20	0.63
Smoking,%	65	30	0.042
Previous angina,%	20	70	0.0006
Prior MI,%	9	0	0.60
*Current disease*			
Time to treatment, hrs	3.5 (2.5-5.0)	7.0 (4.0-9.0)	0.003
Max. QRS duration, ms	90 (80–100)	100 (93–120)	0.60
EF,%	61 (50–72)	56 (45–63)	0.003
Killip-Kimball class >1,%	3	64	0.0001
*Angiographic parameters*			
>1 vessel affected,%	47	90	0.016
TIMI (0–2) before PCI,%	93	100	1.00
TIMI (0–2) after PCI,%	32	60	0.091
CTFG	24 (30–32)	25 (31–100)	0.019
MBG (0–1),%	32	60	0.091
Abciximab,%	26	70	0.008
PCI complications,%	5	40	0.009
*Laboratory findings*			
CPK, U/l	1649 (740–2699)	3473 (2544–4885)	0.011
CK-MB, U/l	159 (62–273)	371 (271–520)	0.014
cTnI (μg/l)	16 (3–45)	7 (1–30)	0.62
Total cholesterol, mmol/l	5.9 (5.2-7.2)	6.2 (5.6-6.8)	0.78
LDL cholesterol, mmol/l	3.9 (3.2-4.8)	3.9 (3.4-4.4)	0.93
HDL cholesterol, mmol/l	1.4 (1.2-1.7)	1.3 (1.1-1.7)	0.64
Triglycerides, mmol/l	1.5 (1.1-2.2)	1.6 (0.9-2.6)	0.93
Glucose, mmol/l	6.4 (5.6-7.7)	9.3 (7.3-14.1)	0.002
CRP (mg/l)	2.2 (0.7-6.3)	2.0 (1.3-6.0)	0.74
Creatinine, μmol/l	84 (73–96)	94 (74–127)	0.26
IL-6, pg/ml	2.0 (0.1-9.0)	8.5 (2.5-20.0)	0.032
sIL-6R, ng/ml	53.0 (41.6-66.2)	51.1 (32.9-65.1)	0.58

*Abbreviations*: *BMI* body mass index, *CK-MB* creatine kinase-myocardial band isoform, *CPK* creatine phosphokinase, *CRP* C-reactive protein, *CTFC* corrected TIMI frame count, *EF* ejection fraction, *HDL* high density lipoprotein, *IL-6* interleukin-6, *IL-6sR* interleukin-6 soluble receptor, *LDL* low density lipoprotein, *MBG* myocardial blush grade, *TIMI* thrombolysis in myocardial infarction scale.

Comparisons were made between patients who survived and those who died within 30 days after STEMI. Values are the medians (and interquartile ranges) or percentages.

The differences observed were not unexpected and included rather predictable parameters, such as age, time to treatment, long-term multiple-vessel CAD, high Killip-Kimball class, higher CPK, in-hospital events, and complicated PCI [20]. Interestingly, the levels of IL-6 at admission were significantly higher in patients who subsequently died compared with those who survived. Concentrations of sIL-6R did not differ between the groups.

**Figure 1:**
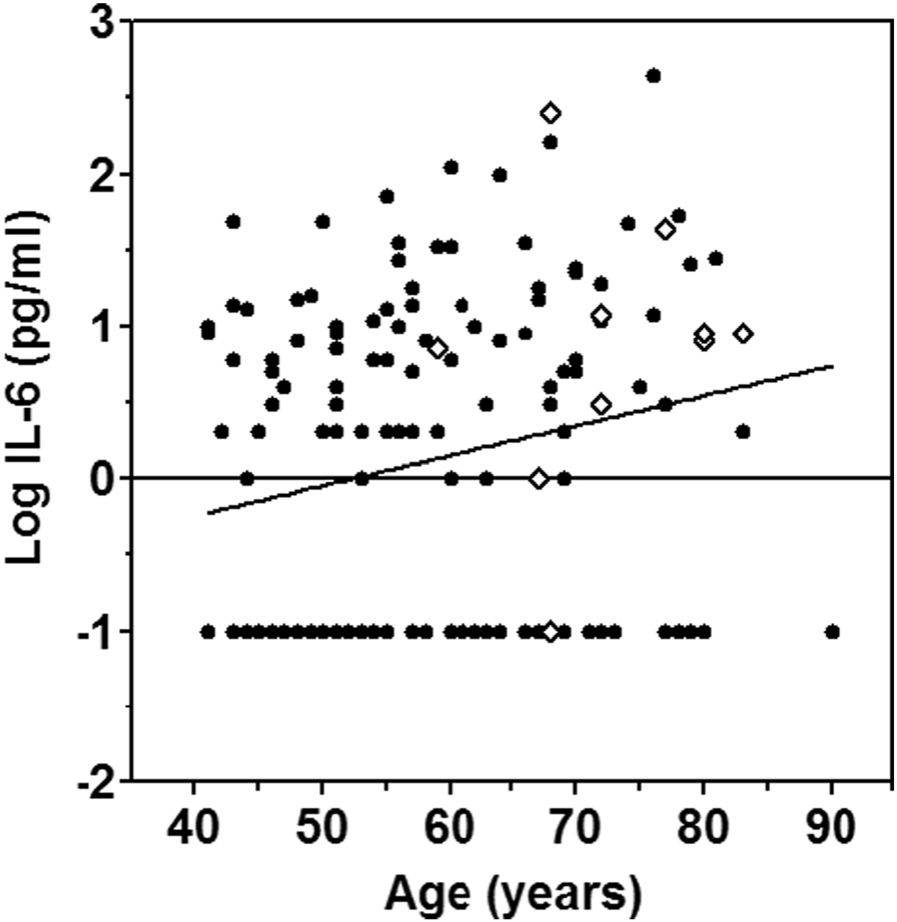
**Correlation between age and serum log [IL-6] concentration.** Survivors – closed symbols; non-survivors – open symbols.

The above data suggested that high IL-6 concentrations might be predicting early mortality in STEMI. However, patients who died were also significantly older (57.8 ± 0.9 vs. 72.6 ± 2.3 years). Serum IL-6 levels are known to increase with age [18] and, indeed, such a correlation was evident also in our patient population (Spearman r = 0.2047, p = 0.009; Figure 1). Therefore, the difference in IL-6 observed had to be corrected for age in order to determine whether it could be specifically related to patients’ outcome. To this end, a regression analysis was performed using age and outcome (treated as a dichotomous variable with “0” and “1” for survivors and non-survivors, respectively) as the predictors. Values of log [IL-6] were predicted significantly in this model (F_2,155_ = 4.72, p = 0.01). The standardized beta-coefficient for age was positive and significant (p = 0.042), whereas that for outcome was positive (higher in those who died) but not significantly so (p = 0.139) (Table 2). In addition, even though CAD was 2.6x more prevalent in patients who died than in those who survived (P = 0.014, t-test), incorporating prior duration of CAD into the regression equation showed that it was not a significant predictor (P = 0.54) and the results were, essentially, unchanged.

**Table 2:** **Predictors of serum log [IL-6] in STEMI**

**Model**		**Unstandardized coefficients**		**Standardized coefficients**	**t**	**Significance**
		**B**	**Std. Error**	**Beta**		
1	Constant	-.841	.457		−1.839	.068
	Age	.016	.008	.169	2.049	.042
	Outcome	.521	.350	.123	1.487	.139

Dependent Variable: Log [IL6].

Regression analysis was performed using age and outcome [survived vs. died] as the predictors.

To confirm the results obtained, we applied a different method of correcting for age. For this analysis only those survivors were selected (n = 17), who were of the same age as those who died (59, 67, 68, 72, 77, 80 and 83 years). This approach made the two groups more similar in age and number (Table 3). Levels of IL-6, log [IL-6] and sIL-6RS in these groups were not at all significantly different.

**Table 3:** **Comparison of survivors and non-survivors of similar age**

	**Survivors (n = 17)**	**Non-survivors (n = 10)**	**P**
Age, yrs	69.1 ± 1.8	72.6 ± 2.3	0.25
Log [IL-6], pg/ml	0.46 ± 0.28	0.84 ± 0.28	0.31
sIL-6R, ng/ml	51.6 ± 4.5	50.5 ± 4.9	0.86

Only survivors of the same age as non survivors were analysed. Values are presented as means ± SE.

Taken together, these results imply that IL-6 concentration had been significantly affected by patients’ age but not independently by patients’ outcome.

## Discussion

Increased levels of IL-6 found in the elderly reflect predominantly an increased prevalence of cardiovascular risk factors or subclinical cardiovascular abnormalities, and the presence of other age-related diseases. Nevertheless, the rise in IL-6 with age is seen even after adjusting for these variables, which may suggest there exists an age-associated dysregulation of IL-6 production [18,19,21,22]. Therefore, the interpretation of increased IL-6 concentrations in the elderly in the context of CAD may be difficult. On the one hand, age-related increase in pro-inflammatory cytokines may pave the way for the disease; on the other hand it may blur a disease-specific increase in cytokine release.

Older individuals with low risk profile might have been under-represented in some studies. When, however, the elderly population is more specifically targeted, the IL-6 assessment appears to improve long-term prediction of future CAD events [23]. It needs to be determined whether the same holds true for early mortality after STEMI. Although our observations indicate it might not always be the case, more well-powered studies would be required.

Including the prior duration of CAD in the regression calculation showed that it was not a significant predictor of patient outcome. Furthermore, we chose to analyse only patients with no history of previous therapy with statins as they are known to damp inflammation and decrease IL-6 release [24,25]. It would be interesting to determine whether earlier exposure to statins impacts on the value of IL-6 (or the lack of it) in predicting early mortality in patients with STEMI treated with primary PCI.

It is accepted that, because of the small group of patients who died (N = 10), care must be taken with interpreting the results, whether they are statistically significant (when a larger sample is required to confirm this difference) or non-significant (when a Type II error might be present). Therefore, any interpretation of the present results must be considered speculative, and need to be confirmed in a larger set of patients.

For the moment, the results of our small single-centre study suggest that high IL-6 levels observed at baseline in non-survivors are related more to their advanced aged rather than point independently to an increased risk of early death. It is accepted that percutaneous coronary intervention (PCI) and coronary artery bypass grafting (CABG) are both good options for patients with advanced CAD [26]. Nevertheless, the challenge of finding optimal markers of long-term prognosis in such patients remains. The present study indicates that levels of IL-6 do not seem to be sufficiently disciminatory in this regard.

## Abbreviations

ACS: Acute coronary syndromes
BMI: Body mass index
CAD: Coronary artery disease
CK-MB: Creatine kinase-myocardial band isoform
CPK: Creatine phosphokinase
CRP: C-reactive protein
CTFC: Corrected TIMI frame count
EF: Ejection fraction
HDL: High density lipoprotein
IL-6: Interleukin-6
IL-6R: IL-6 receptor
LDL: Low density lipoprotein
MBG: Myocardial blush grade
MI: Myocardial infarction
PCI: Percutaneous coronary intervention
sIL-6R: Soluble IL-6 receptor
STEMI: ST-elevation myocardial infarction
TIMI: Thrombolysis in myocardial infarction scale
